# Association between creative self-efficacy and innovation literacy among undergraduate nursing students: a correlation analysis

**DOI:** 10.3389/fpubh.2026.1815593

**Published:** 2026-05-28

**Authors:** Ruiqi Lyu, Zhenhong Zhou, Yaliqin Wang, Mengya Xu, Ni Zhang, Fenglin Xu, Jiaxin Wang, Ziang Chu, Hongdan Li, Jianghua Wu

**Affiliations:** 1 School of Nursing, Shandong First Medical University and Shandong Academy of Medical Sciences, Taian, Shandong, China; 2 Center for Nuclear Medicine and Molecular Imaging, Shandong Cancer Hospital and Institute, Shandong First Medical University and Shandong Academy of Medical Sciences, Jinan, China

**Keywords:** active learning state, creative self-efficacy, innovation literacy, social capital, undergraduate nursing students

## Abstract

**Background:**

Healthcare professionals frequently encounter unanticipated workplace challenges, positioning innovation literacy as an essential competency for career advancement. As future practitioners, undergraduate nursing students require adequate innovation literacy to support the optimization of clinical nursing practice and nursing education. This study aimed to explore the association between creative self-efficacy and innovation literacy, and to investigate the potential sequential correlational roles of active learning state and social capital in this relationship.

**Methods:**

A cross-sectional questionnaire survey was conducted among 337 undergraduate nursing students using internationally validated psychometric scales. Hierarchical regression and the PROCESS macro (Model 6) were applied to examine a sequential correlational framework, while demographic variables were controlled for as potential con founders.

**Results:**

The hypothesized sequential correlational framework was statistically supported. Creative self-efficacy showed a positive correlation with innovation literacy through a direct path (*β* = 0.315) and via two distinct sequential indirect links. The first path involved active learning state only (indirect link = 0.221), accounting for 33.48% of the total association. The second sequential path proceeded from active learning state to social capital (indirect relation = 0.080), accounting for 12.12% of the total association. Additionally, social capital showed an independent correlational path (indirect link = 0.044, contributing 6.67% to the total association). The overall framework accounted for 68.6% of the variance in innovation literacy.

**Conclusion:**

This study identified notable correlational links between creative self-efficacy and innovation literacy among undergraduate nursing students. Active learning state and social capital functioned as sequential intermediate variables within this correlational relationship. The findings suggest that targeted cultivation of creative self-efficacy, together with facilitating active learning and social capital accumulation, is associated with higher innovation literacy. These findings point to the potential value of educational strategies that foster these factors in relation to innovation literacy development.

## 1 Introduction

According to the Global Strategy on Human Resources for Health, the global health workforce is projected to face a shortage of 18 million health professionals by 2030 (1). Meanwhile, population aging in China continues to accelerate, placing substantial pressure on healthcare systems and necessitating service efficiency optimization under workforce constraints. Although artificial intelligence (AI) is applied in the management of complex clinical scenarios, its integration into nursing practice also presents unique professional challenges for frontline nurses (2). As prospective nursing professionals, nursing students must develop innovative competencies that are related to addressing evolving global health challenges (3). In this context, innovation literacy is a professional competency linked to high-quality modern healthcare delivery. Accordingly, innovation literacy has become an indispensable core competency for nursing professionals, and its cultivation showed links to individual internal psychological factors.

According to Bandura (1997), creative self-efficacy is an individual-level psychological belief, referring to individuals’ subjective judgments on whether they can successfully accomplish innovation-related tasks (4). As a cognition-level construct, it reflects future-oriented subjective assessments. In the context of nursing education, self-efficacy serves as a key psychological resource for nursing students to evaluate their career choices, adapt to the rapidly changing healthcare environment, and achieve professional growth (5). Although numerous empirical studies have identified the relationship between creative self-efficacy and innovation-related behavior (6–8), research on the association between creative self-efficacy and innovation literacy—a more comprehensive competency construct—remains insufficient. Notably, as an overt behavioral outcome, focusing solely on innovation-related behavior provides limited insight into the developmental mechanisms of innovation competence. Therefore, to effectively address global health challenges, nursing education may benefit from cultivating nursing students’ innovation-related behaviors while exploring the psychological mechanisms and functional pathways underlying the formation of innovation literacy. In addition to individual internal beliefs, external social resources in individuals’ environments are associated with learning and innovation processes, among which social capital shows particularly strong associations.

Social capital encompasses three core dimensions: (1) cross-group interpersonal connections, (2) social cohesion, and (3) civic engagement (9). As a structural resource, social capital shows correspondence with goal formation through the provision of information, opportunities, and supportive networks. In a cross-sectional study of 2,247 Japanese university students, social capital (including interpersonal relationships, mutual trust, and social bonds) was related to higher levels of academic motivation, reflecting an autonomous intrinsic motivational factor (10). Furthermore, social capital was associated with individuals’ learning capabilities, which covaried with innovation in product upgrades, process re-engineering, and management model optimization (11). Nursing students navigate complex interpersonal dynamics, including peer relationships and future physician-patient partnerships. Their future practice requires professional knowledge and adaptability to complex disease trajectories. Within the interactive environment supported by social capital, the learning approaches adopted by individuals also demonstrated strong associations with the cultivation of innovative literacy, among which proactive learning demonstrated significant procedural associations.

Active learning state (ALS) refers to the tendency of learning initiative, bidirectional collaborative interaction, sustainability, and non-judgmental feedback (12). Proactive learning is defined as a dynamic, process-oriented characteristic reflecting the manner and quality of learning behaviors, rather than learning outcomes or competencies themselves. Studies indicate that active learning approaches showed relations with competencies among health professions students, including collaboration with healthcare professionals and care provision in complex patient situations (13). A systematic review of 33 empirical studies found ALS significantly related to undergraduates’ clinical problem-solving competencies and showed links to key capabilities such as communication and innovation (14). Notably, a nationwide survey of 1,241 medical students found positive correspondence among participation in innovation and entrepreneurship programs, ALS levels, and creative ideation metrics (15). A study focusing on the pathology curriculum demonstrated that active learning strategies aligned with student engagement and critical thinking (16). However, Qian et al. (17) reported a link between deep active learning experiences and beliefs in creative abilities among undergraduate health professions students. The associations among active learning state, innovation literacy, and creative self-efficacy remain under debate. This study further explores these relationships. Based on the above analysis of internal beliefs, external resources, and learning processes, innovation literacy was linked to multiple factors, requiring a more systematic definition of its connotation and structure.

Innovation literacy is a comprehensive construct at the level of competency performance, referring to individuals’ ability to transform innovative cognition into actual innovative outcomes, including knowledge application, technological adaptation, and innovative practice (18). Innovation literacy targets behavioral-level abilities and reflects objective assessments of current capabilities. In the era of information technology, nurses’ access to and application of nursing informatics showed links to nursing decision-making quality (19). In current nursing education, nursing students’ innovative competence encompasses familiarity with contemporary technologies and the ability to develop approaches to related issues (20). These observations coincided with efforts to prepare nursing students in higher education to adapt to rapidly developing digital advancements. Taken together, current findings aligned with discussions on a shift in higher education: a perspective shifting from knowledge transmission toward the cultivation of creative abilities through psychological and instructional support mechanisms (21). Although numerous studies have documented relations between instructional reforms and innovation literacy among students (21), empirical evidence focused on innovation literacy development among undergraduate nursing students remains limited. Against this background, this study, grounded in the Socialization-Externalization-Combination-Internalization (SECI) model, explores factors and their connections with innovation literacy among undergraduate nursing students.

## 2 Theoretical framework

### 2.1 Core mechanisms of the SECI model and its applicability at the individual level

The SECI model, originally proposed by Nonaka, comprises four stages: Socialization, Externalization, Combination, and Internalization, describing the dynamic conversion cycle between tacit and explicit knowledge (22). Although this model was initially applied in the field of organizational knowledge creation, its analytical framework is also applicable across different levels, emphasizing that knowledge creation originates from the sharing and transformation of individual tacit knowledge. Organizational-level knowledge creation is closely connected with individual cognitive activities; stages such as socialization and externalization are reflected at the individual level as processes of interaction and reflection. Therefore, mapping the four SECI stages onto individual-level psychological processes should not be regarded as a mechanical application of organizational theory, but can be understood as an extension based on the theoretical premise that “knowledge creation begins with the individual.

### 2.2 Theoretical mapping of the four variables onto SECI stages

The core logic of this study is that the formation of individual innovative competence follows the knowledge transformation sequence described by SECI. The four key variables can be mapped onto different stages of the SECI cycle, with specific mapping relationships and theoretical foundations as follows.

#### 2.2.1 Creative self-efficacy → socialization stage

Creative self-efficacy is linked to individuals’ engagement in social interaction, during which they accumulate innovation-related tacit experiences associated with the socialization stage (23). This mechanism aligns with the socialization stage of the SECI model, which emphasizes the transmission of tacit knowledge through shared experiences, imitation, and informal interaction. In this study, creative self-efficacy is understood as a psychological prerequisite related to the initiation of the socialization process. Individuals with high creative self-efficacy tend to actively seek interpersonal interaction, a process connected with the accumulation of innovation-related tacit experiences and the activation of the SECI cycle.

#### 2.2.2 Active learning state → externalization Stage

The core of the externalization stage is the transformation of tacit knowledge into transmittable explicit knowledge, with key mechanisms including reflection, expression, conceptualization, and dialogue. Students with active learning tendencies are more inclined to participate in peer discussions, raise questions, clarify ambiguous concepts, and articulate personal insights in communicable forms (24, 25). These behaviors are associated with the transformation of tacit knowledge into explicit knowledge. In other words, active learning state provides a behavioral foundation related to the externalization of internal understanding, a process corresponding to the transition of knowledge forms from tacit to explicit.

#### 2.2.3 Social capital → combination stage

The combination stage emphasizes the integration of explicit knowledge from diverse sources into systematic cognitive frameworks. Social capital, as structural resources accumulated through collaboration, feedback, and diverse relational networks, can be understood as a contextual condition associated with the combination process (26). For example, in collaborative learning situations, students pool, compare, and integrate their externalized knowledge to form structured knowledge systems (25). Social capital functions as structural support in this process: it is linked to channels of knowledge acquisition, arenas for integration, and foundations of collaborative trust. This understanding aligns with the functional positioning of “enabling rather than substituting.”

#### 2.2.4 Innovation literacy → internalization stage

The core mechanism of the internalization stage is the re-transformation of systematic explicit knowledge into individual tacit capabilities through practical application and reflective experience. Innovation literacy can be understood as an outcome connected with this process: individuals internalize externally acquired knowledge and skills into stable, autonomously applicable innovative capabilities through repeated practice and reflection (18). Innovation literacy differs from simple accumulation of external knowledge, manifesting as individual traits formed through the complete SECI cycle, reflecting the transformation from “knowing what” to “being able to do.

### 2.3 Existing evidence of the SECI model in education and theoretical positioning of this study

The application of the SECI model in the educational field, particularly in fostering innovative competence, has received partial empirical support. Research indicates that university students’ learning outcomes are linked to enhanced self-learning capabilities and increased interactive value, a process corresponding to creativity growth (27). Different socialization levels contribute distinct forms of new knowledge, influencing knowledge generation and flow efficiency, a process associated with final innovative capability (26). Theoretically, socialization provides opportunities for learners to access diverse perspectives, a process connected with creative stimulation; externalization involves articulating thoughts clearly; combination is related to integrating diverse information sources, a process linked to cognitive boundary expansion; internalization corresponds to the integration of new insights into personal skill systems (28).

However, some studies have noted that the effects of the SECI model on innovative knowledge transformation are not stable (29), partly due to: (1) most studies examined only a single dimension of SECI rather than assessing the complete four-stage cycle (28); (2) the acquisition of innovation literacy may depend on contextual factors such as learning atmosphere, motivation, and knowledge resources (30, 31); (3) methodological differences lead to inconsistent results. These inconsistencies highlight the necessity of establishing a complete chain model and conducting rigorous examination, rather than assuming that SECI has universal, unidirectional positive effects.

### 2.4 Theoretical contribution and research hypotheses of this study

**Figure 1:**
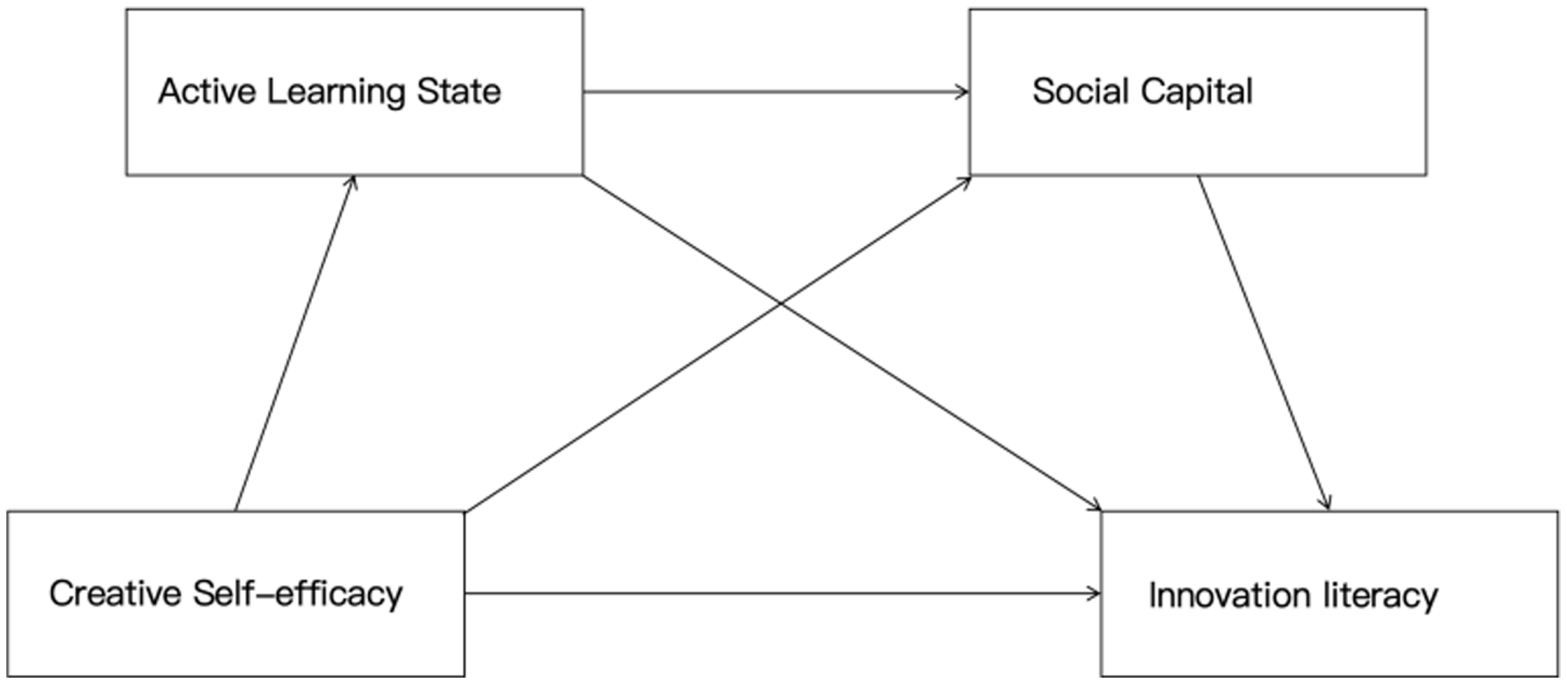
A proposed sequential framework describing the intermediate associations of active learning state and social capital in the association between creative self-efficacy and innovation literacy.

Based on the above analysis, this study constructs the four SECI stages into an empirically testable chain mediation model, aiming to examine the theoretical sequential relationships among functional nodes in cross-sectional data, laying groundwork for subsequent longitudinal validation research. The sequence of the four stages is built upon the internal logic of knowledge form transformation: socialization (corresponding to tacit experience acquisition) → externalization (corresponding to explicit knowledge encoding) → combination (corresponding to multi-source knowledge integration) → internalization (corresponding to stable capability formation). Creative self-efficacy, active learning state, social capital, and innovation literacy are mapped onto the functional nodes of this sequence, respectively. This model differs from mechanically “fitting” variables into SECI dimensions; it reflects theoretical sequencing derived from the knowledge transformation logic of SECI and the functional positioning of each variable. This sequence addresses the limitation of previous studies that neglected the complete cycle and is aligned with the design of stage-specific intervention strategies in nursing education (see Figure 1). Based on the above theoretical derivation, this study proposes the following hypotheses:

H1: Innovative self-efficacy is positively statistically associated with active learning state, social capital, and innovation literacy in sequential order.

H2: Innovative self-efficacy is positively statistically associated with innovation literacy.

H3: Active learning state and social capital sequentially mediate the positive statistical association of Innovative self-efficacy with innovation literacy.

## 3 Method

### 3.1 Design

This study explores the multidimensional and synergistic statistical associations underlying innovation literacy among undergraduate nursing students. Additionally, it examines patterns relevant to nursing educators in the context of undergraduate nursing students’ innovation-related competencies. This study used a descriptive cross-sectional design. Data were collected from undergraduate nursing students at a single medical university using structured questionnaires.

### 3.2 Participants

The sampling frame included all undergraduate nursing students enrolled at a medical university during the 2023–2024 academic year. Recruitment announcements were posted via the campus website, and the Questionnaire Star link was distributed to class WeChat groups by grade counselors. Students arranged their own questionnaire completion time and voluntarily participated after providing informed consent. The sampling period was from December 2023 to March 2024. Regarding common method bias, this study implemented the following procedural measures: Anonymous completion: the questionnaire did not collect names, student IDs, or other identifiable information, a measure associated with reduced social desirability effects. Staggered distribution: questionnaires were distributed in multiple batches through online and offline channels over a four-month period, with students selecting their own completion time, allowing for greater situational diversity. Differentiated instructions: different scales employed different instructions and wording formats, to reduce the likelihood of fixed response patterns. Quality control: lie-detection items and attention-check questions were embedded, to screen for careless responding. According to Kendall’s sample size estimation principle, the number of observations should be at least 10 times the number of independent variables (32). Considering an anticipated non-response rate of approximately 20% in this study, the minimum required sample size was calculated to be 276. A total of 400 questionnaires were distributed, and 370 were recovered, with a recovery rate of 92.5%. After excluding 33 invalid questionnaires (completion time < 3 min, regularized response patterns, or failure of lie-detection items), 337 valid questionnaires were obtained, yielding an effective rate of 91.1% and a response rate of 84.3%. The final sample size met the minimum requirement calculated by Kendall’s principle.

#### 3.2.1 Inclusion criteria

1. Currently enrolled undergraduate students in nursing science programs at the selected medical university.
2. Capacity for independent questionnaire completion with signed informed consent.

#### 3.2.2 Exclusion criteria

1. Declined participation or inability to complete the questionnaire in full.
2. Absence from school during the sampling period due to medical leave, military service, academic suspension, or other reasons.

### 3.3 Data collection tools

#### 3.3.1 Student demographic information questionnaire

The items included in this questionnaire were adapted from previously published research instruments. It was used to collect participants’ demographic characteristics, including age, sex, ethnicity, academic grade, place of origin, political affiliation, and prior experience as a class cadre.

#### 3.3.2 Creative self-efficacy scale for college students

The Creative Self-Efficacy Scale for College Students assesses creative self-efficacy in this population (33). This scale includes 21 items and has four dimensions: sensitivity, flexibility, originality, and fluency. Each item was rated on a 5-point Likert scale (1 = not at all, 2 = unlikely, 3 = moderately likely, 4 = more likely, and 5 = entirely feasible). The total score for this scale ranges from 21 to 105. In the present sample (*N* = 337), Cronbach’s *α* = 0.967 (dimensions: 0.886–0.904); χ^2^/df = 2.18, CFI = 0.960, TLI = 0.954, RMSEA = 0.059 (90% CI: 0.051–0.067), SRMR = 0.029. Composite reliability (CR) ranged from 0.89 to 0.92, average variance extracted (AVE) from 0.67 to 0.68, and standardized factor loadings from 0.70 to 0.95, all *p* < 0.001. See Tables 1, 2.

**Table 1:** The Cronbach’s *α* of scales.

Variable	Dimension	Items	Cronbach’s α	
Creative self-efficacy	Sensitivity	6	0.904	0.967
	Flexibility	6	0.897	
	Originality	5	0.886	
	Fluency	4	0.877	
Active learning state	Learning motivation	5	0.714	0.935
	Learning objectives	4	0.753	
	Learning depth	4	0.804	
	Control learning	4	0.825	
	Consolidation	4	0.845	
Social capital	Strong connection	3	0.869	0.871
	Weak connection	4	0.941	
Innovation literacy	Innovation ability	6	0.904	0.966
	Learning ability	4	0.874	
	Innovation knowledge	3	0.852	
	Innovation motivation	4	0.860	
	Innovation will	3	0.816	

**Table 2:** The confirmatory factor analysis indicators of each scale.

	Structural validity						Convergent validity			
Variable	X^2^/df	CFI	TLI	RMSEA	90% CI	SRMR	CR	AVE	Standardized factor loadings	*P*
Creative self-efficacy	2.18	0.960	0.954	0.059	0.051–0.067	0.029	0.89–0.92	0.67–0.68	0.70–0.95	<0.001
Active learning state	2.76	0.915	0.900	0.074	0.067–0.082	0.049	0.78–0.85	0.372–0.610	0.557–0.797	<0.001
Social capital	2.18	0.975	0.956	0.065	0.032–0.098	0.027	0.62/0.78	0.46/0.54	0.52–0.88	<0.001
Innovation literacy	3.53	0.949	0.940	0.070	0.058–0.082	0.038	0.816–0.904	0.568–0.655	0.686–0.832	<0.001

#### 3.3.3 The measure of active learning state

The Measure of Active Learning State for Healthcare Undergraduates assesses active learning state in this population (34). The scale includes 21 items and comprises five dimensions: learning motivation, learning objectives, learning depth, control learning, and consolidation. The scale has 17 positive items. Each item is rated on a 5-point scale (5 = very good, 4 = good, 3 = fair, 2 = poor, and 1 = very poor). It also contains four negative items. Each item is rated on a 5-point scale (1 = very bad, 2 = poor, 3 = generally, 4 = good, 5 = very good). The total score for this scale ranges from 21 to 105. In the present sample (N = 337), Cronbach’s *α* = 0.935 for the overall scale and 0.714–0.845 for the five dimensions. Confirmatory factor analysis yielded acceptable fit: χ^2^/df = 2.76, CFI = 0.915, TLI = 0.900, RMSEA = 0.074 (90% CI: 0.067–0.082), SRMR = 0.049. Composite reliability (CR) ranged from 0.78 to 0.85 and average variance extracted (AVE) from 0.372 to 0.610 (core dimensions ≥ 0.549); standardized factor loadings were significant (*p* < 0.001) and ranged from 0.557 to 0.797, meeting the conventional thresholds for convergent and discriminant validity. See Tables 1, 2.

#### 3.3.4 Social capital inventory

The measure of social capital inventory was developed by Gao (35), who improved this scale. This scale includes seven items and assesses two factors: strong connection and weak connection. Each item is rated on a 5-point Likert scale (1 = disagree, 2 = somewhat disagree, 3 = generally, 4 = somewhat agree, 5 = agree). The total score for this scale ranges from 7 to 35. Weak questionnaire design has been associated with non-response bias (36). To address potential associations with response quality, we slightly revised the wording of four items to better fit undergraduate nursing students. Specifically, we replaced “government personnel” with “teachers and counselors”, “customers, suppliers, and retailers” with “classmates and roommates”, “financial institution staff” with “medical staff and preceptors”, and “personnel from other enterprises and commercial groups” with “patients and their families”, resulting in a questionnaire tailored to nursing students. In the present sample (N = 337), Cronbach’s *α* = 0.871 for the overall scale and 0.869–0.941 for the two dimensions. Confirmatory factor analysis yielded satisfactory fit: χ^2^/df = 2.18, CFI = 0.975, TLI = 0.956, RMSEA = 0.065 (90% CI: 0.032–0.098), SRMR = 0.027. Composite reliability (CR) was 0.62 for “strong connection” and 0.78 for “weak connection”; average variance extracted (AVE) was 0.46 and 0.54, respectively. Standardized factor loadings ranged from 0.52 to 0.88 (all *p* < 0.001), supporting convergent and discriminant validity. The CR value for the strong connection dimension was 0.62, close to the conventional threshold of 0.7. Combined with the AVE value of 0.46 and significant standardized factor loadings ranging from 0.52 to 0.88, the adapted scale still satisfies the basic criteria for convergent validity. See Tables 1, 2.

#### 3.3.5 The measure of innovation literacy

Developed by Yang (37), this scale was originally designed for postgraduates’ innovation literacy. This scale includes 22 items and has five dimensions: innovation ability, learning ability, innovation knowledge, innovation motivation, and innovation will. The scale includes two polygraph items. Each item is rated on a 5-point Likert scale (1 = very inconsistent, 2 = somewhat inconsistent, 3 = average, 4 = somewhat consistent, 5 = very consistent). The total score for this scale ranges from 22 to 110. In the present sample (*N* = 337), Cronbach’s *α* = 0.966 for the overall scale and 0.816–0.904 for the five dimensions. Confirmatory factor analysis was performed using the robust maximum likelihood (MLR) estimation method. The model demonstrated acceptable fit:χ^2^/df = 3.53, Robust CFI = 0.949, Robust TLI = 0.940, Robust RMSEA = 0.070 (90% CI: 0.058–0.082), SRMR = 0.038. Composite reliability (CR) ranged from 0.816 to 0.904, and average variance extracted (AVE) ranged from 0.586 to 0.655, with standardized factor loadings ranging from 0.686 to 0.832 (all *p* < 0.001). These values meet the recommended criteria for good reliability, convergent validity, and discriminant validity. These properties are consistent with the requirements for subsequent correlational analysis (see Tables 1, 2).

### 3.4 Data collection

Participants meeting the inclusion criteria provided written informed consent. Data were collected via the Questionnaire Star platform, with standardized verbal instructions provided to explain the purpose and completion requirements. Investigators were available to address any issues during questionnaire completion. Upon completion, investigators checked questionnaires for completeness. Those with missing key information or patterned responses were excluded. Throughout the data collection process, measures were implemented to ensure confidentiality, objectivity, and scientific rigor, with attention to data quality.

### 3.5 Data analysis

This study used IBM SPSS Statistics 26.0 for all statistical analyses. First, descriptive statistics were used to describe the demographic characteristics of undergraduate nursing students and report scores for the Creative Self-Efficacy Scale, the Active Learning State Scale, the Social Capital Inventory, and the Innovation Literacy Scale. Categorical variables were described using frequencies and percentages, whereas continuous variables were presented as means and standard deviations (SD) if normally distributed, or as medians (M) and interquartile ranges (IQR) if non-normally distributed. For normally distributed continuous variables with homogeneous variance, independent samples t-tests and one-way ANOVA were conducted. Sex, grade, residence, and class leader status were adjusted for as potential confounders in all analyses.

Prior to regression analysis, Pearson’s product–moment correlation analysis was performed to examine the bivariate associations among all study variables. Subsequently, hierarchical regression was conducted to examine the independent associations of creative self-efficacy, active learning state, and social capital with innovation literacy.

To examine the mediating associations of the variables, the PROCESS macro embedded in IBM SPSS Statistics 26.0 was used, and a corresponding mediation model was constructed. In the mediation analysis, sex, grade, residence, and class leader status were included as covariates. A bootstrapping procedure was implemented to examine the sequential associations involving active learning state and social capital in the context of the association between CSE and innovation literacy among undergraduate nursing students. The bootstrapping sample size was set to 5,000, and 95% bias-corrected confidence intervals (CIs) were calculated. Consistent with standard statistical conventions, a mediating association showed statistical significance if the 95% CI excluded zero.

## 4 Results

### 4.1 General information of nursing students

A total of 337 undergraduate nursing students were investigated in this study, including 73 males (21.7%) and 264 females (78.3%). Among respondents, 172 (51%) were under 20, and 165 (48.9%) were 20 years or older. Most respondents were Han (331; 98.2%), and 6 were ethnic minorities (1.8%). Eighty-eight students (26.1%) were in the first year, 73 (21.7%) in the sophomore year, 111 (32.9%) in the junior year, and 65 (19.3%) in the senior year. One hundred and ninety-nine students (59.1%) lived in rural areas and 138 (40.9%) in urban areas. Most respondents were Communist Youth League members, followed by the masses: 195 were Communist Youth League members (57.9%), 72 were mass members (21.4%), and 36 were probationary party members (10.7%). Thirty-four (10.1%) were CPC members. Among respondents, 140 (41.5%) were class leaders, and 197 (58.5%) were not. Other details are shown in Table 3.

**Table 3:** Descriptive statistics of the general demographic information of undergraduate nursing students (*N* = 337).

Variables	Categories	*N*	*N* (%)
Age	≤20	172	51.0
	>20	165	48.9
Sex	Male	73	21.7
	Female	264	78.3
Nation	The Han nationality	331	98.2
	Minority	6	1.8
Grade	Freshman	88	26.1
	Sophomore	73	21.7
	Junior	111	32.9
	Senior	65	19.3
Residence	Town	138	40.9
	Country	199	59.1
Political status	Member of the communist party of China	34	10.1
	Probationary party members	36	10.7
	League member	195	57.9
	Masses	73	21.4
Class leader status	Yes	140	41.5
	No	197	58.5

### 4.2 Correlation analysis of each study variable

The correlation analysis of CSE, innovation literacy, active learning state, and social capital, including their dimensions, is shown in Table 4. The mean scores for innovative self-efficacy (73.04 ± 15.10), active learning state (74.63 ± 13.37), social capital (22.00 ± 5.71), and innovation literacy (72.18 ± 13.63) all exceed the respective theoretical midpoints of their scales. Based on the results, innovation literacy, CSE, active learning state, and social capital were positively correlated. Innovation literacy is significantly positively correlated with CSE, active learning state, and social capital (*p* < 0.05). Therefore, H1 is true.

**Table 4:** Correlation analysis between study variables for undergraduate nursing students (*N* = 337).

Variable	M (SD)	Creative self-efficacy	Sensitivity	Flexibility	Originality	Fluency	Active learning state	Learning motivation	Learning objectives	Learning depth	Control learning	Consolidation	Social Capital	Strong connection	Weak connection	Innovation literacy	Innovation ability	Learning ability	Innovation knowledge	Innovation motivation	Innovation will
Creative self-efficacy	3.48 (0.72)	1																			
Sensitivity	3.51 (0.77)	0.934^**^	1																		
Flexibility	3.46 (0.75)	0.956^**^	0.859^**^	1																	
Originality	3.48 (0.76)	0.947^**^	0.832^**^	0.871^**^	1																
Fluency	3.46 (0.77)	0.918^**^	0.780^**^	0.848^**^	0.871^**^	1															
Active learning state	3.55 (0.64)	0.607^**^	0.560^**^	0.590^**^	0.574^**^	0.557^**^	1														
Learning motivation	3.60 (0.67)	0.518^**^	0.503^**^	0.503^**^	0.469^**^	0.461^**^	0.855^**^	1													
Learning objectives	3.51 (0.76)	0.564^**^	0.508^**^	0.548^**^	0.535^**^	0.533^**^	0.838^**^	0.707^**^	1												
Learning depth	3.39 (0.77)	0.924^**^	0.815^**^	0.952^**^	0.875^**^	0.821^**^	0.586^**^	0.477^**^	0.544^**^	1											
Control learning	3.59 (0.78)	0.546^**^	0.499^**^	0.538^**^	0.517^**^	0.496^**^	0.871^**^	0.635^**^	0.609^**^	0.545^**^	1										
Consolidation	3.67 (0.74)	0.494^**^	0.451^**^	0.476^**^	0.482^**^	0.445^**^	0.871^**^	0.651^**^	0.646^**^	0.505^**^	0.777^**^	1									
Social capital	3.14 (0.82)	0.489^**^	0.438^**^	0.451^**^	0.481^**^	0.476^**^	0.618^**^	0.557^**^	0.530^**^	0.432^**^	0.519^**^	0.501^**^	1								
Strong connection	3.71 (0.85)	0.462^**^	0.436^**^	0.430^**^	0.449^**^	0.420^**^	0.646^**^	0.580^**^	0.541^**^	0.450^**^	0.564^**^	0.602^**^	0.698^**^	1							
Weak connection	2.72 (1.08)	0.371^**^	0.321^**^	0.341^**^	0.369^**^	0.379^**^	0.433^**^	0.392^**^	0.380^**^	0.304^**^	0.351^**^	0.305^**^	0.906^**^	0.330^**^	1						
Innovation literacy	3.61 (0.68)	0.679^**^	0.615^**^	0.632^**^	0.660^**^	0.658^**^	0.741^**^	0.623^**^	0.612^**^	0.622^**^	0.659^**^	0.706^**^	0.664^**^	0.672^**^	0.478^**^	1					
Innovation ability	3.65 (0.71)	0.639^**^	0.575^**^	0.595^**^	0.620^**^	0.623^**^	0.682^**^	0.583^**^	0.540^**^	0.590^**^	0.621^**^	0.660^**^	0.592^**^	0.651^**^	0.396^**^	0.961^**^	1				
Learning ability	3.62 (0.74)	0.636^**^	0.566^**^	0.607^**^	0.615^**^	0.610^**^	0.702^**^	0.590^**^	0.592^**^	0.608^**^	0.615^**^	0.688^**^	0.591^**^	0.617^**^	0.415^**^	0.925^**^	0.860^**^	1			
Innovation knowledge	3.49 (0.76)	0.627^**^	0.583^**^	0.574^**^	0.613^**^	0.596^**^	0.709^**^	0.585^**^	0.575^**^	0.538^**^	0.625^**^	0.652^**^	0.658^**^	0.569^**^	0.532^**^	0.896^**^	0.805^**^	0.807^**^	1		
Innovation motivation	3.60 (0.75)	0.639^**^	0.580^**^	0.586^**^	0.618^**^	0.632^**^	0.677^**^	0.563^**^	0.555^**^	0.590^**^	0.606^**^	0.658^**^	0.635^**^	0.617^**^	0.474^**^	0.927^**^	0.863^**^	0.823^**^	0.803^**^	1	
Innovation will	3.65 (0.77)	0.586^**^	0.530^**^	0.543^**^	0.574^**^	0.564^**^	0.655^**^	0.554^**^	0.583^**^	0.523^**^	0.570^**^	0.588^**^	0.610^**^	0.629^**^	0.432^**^	0.884^**^	0.834^**^	0.751^**^	0.760^**^	0.761**	1

^**^P < 0.01; ^*^P < 0.05.

### 4.3 Univariate variance analysis of innovation literacy among undergraduate nursing students

ANOVA and t-tests were performed for innovation literacy in the general demographic data. Using undergraduate nursing students’ sense of CSE as the observation index, the comparative differences in innovation literacy scores among students with different demographic characteristics were analyzed. Nursing students differed significantly in innovation literacy scores by sex, grade, residence, and class leader status (*p* < 0.05; Table 5).

**Table 5:** Comparison of scores on innovation literacy among undergraduate nursing students with different demographic characteristics (*N* = 337).

Variables	Categories	$\bar{x}$ ±*s*	t/F(p)
Age	≤20	72.640 ± 13.593	0.532
	>20	71.709 ± 13.702	
Sex	Male	75.397 ± 16.052	0.047
	Female	71.296 ± 12.777	
Nation	The Han nationality	72.145 ± 0.751	0.697
	Minority	74.333 ± 5.493	
Grade	Freshman	76.659 ± 13.997	0.001
	Sophomore	68.575 ± 12.027	
	Junior	70.820 ± 14.165	
	Senior	72.508 ± 12.503	
Residence	Town	74.326 ± 14.727	0.019
	Country	70.699 ± 12.72	
Political status	Member of the communist party of China	75.353 ± 13.998	0.500
	Probationary party members	72.944 ± 12.663	
	League member	71.554 ± 13.088	
	Masses	72.014 ± 15.341	
Class leader status	Yes	74.800 ± 13.700	0.003
	No	70.325 ± 13.312	

### 4.4 Hierarchical regression analysis of factors influencing innovation literacy among undergraduate nursing students

To assess multicollinearity, collinearity diagnostics were initially conducted. The results showed that tolerance values ranged from 0.491 to 0.976 (all > 0.1) and Variance Inflation Factor (VIF) values ranged from 1.025 to 2.036 (all < 10), consistent with the absence of serious multicollinearity (see Table 6). Subsequently, hierarchical regression was performed to examine factors associated with innovation literacy. The total score of innovation literacy was used as the outcome variable. Variables were entered into the model in four blocks: Block 1 included control variables (sex, grade, residence, and class leader status); Block 2 added creative self-efficacy (CSE); Block 3 added active learning state (ALS); and Block 4 added social capital (SC). Prior to regression, variable assignment was confirmed (see Table 7). The entry criterion was set to *α* = 0.05 and the removal criterion to *α* = 0.10. The results showed that residence, creative self-efficacy, active learning state, and social capital showed significant independent associations with innovation literacy. The final regression model accounted for 68.6% of the variance in innovation literacy (*R* = 0.828; adjusted *R*^2^ = 0.679). See Table 6.

**Table 6:** Hierarchical multiple regression analysis predicting innovation literacy among undergraduate nursing students (*N* = 337).

Category	Variable	Model 1	Model 2	Model 3	Model 4	Tolerance	VIF
Control variables	Sex	−0.083	−0.030	−0.028	0.004	0.935	1.069
	Grade	−0.116*	−0.059	−0.035	−0.042	0.959	1.043
	Residence	−0.116*	−0.112**	−0.091**	−0.081**	0.971	1.030
	Class leader status	−0.172**	−0.066	−0.037	−0.014	0.921	1.086
Independent variable	Creative self-efficacy	—	0.660***	0.358***	0.315***	0.605	1.652
Mediating variables	Active learning state	—	—	0.508***	0.373***	0.491	2.036
	Social capital	—	—	—	0.270***	0.572	1.749
Model statistics	R^2^	0.065	0.483	0.644	0.686		
	Adjusted R^2^	0.054	0.476	0.638	0.679		
	F	5.783***	61.954***	99.623***	102.704***		
	ΔR^2^	0.065***	0.418***	0.161***	0.042***		
	ΔF	5.783***	268.031***	149.238***	43.751***		

Values presented are standardized regression coefficients (β).

*p < 0.05, **p < 0.01, ***p < 0.001.

**Table 7:** Variable assignment of factors related to innovative literacy among undergraduate nursing students.

Variable	Value
Sex	Male = 1, Female = 2
Grade	Freshman = 1, Sophomore = 2, Junior = 3, Senior = 4
Residence	town = 1, country = 2
Class leader status	Yes = 1, No = 2

### 4.5 Examination of sequential intermediate associations

Sequential intermediate associations were examined using the PROCESS macro (Model 6) in SPSS 26.0, with CSE as the predictor variable, innovation literacy as the outcome variable, and both active learning state and social capital as intermediate variables. Sex, grade, residence, and class leader status were included as control variables. The nonparametric percentile Bootstrap method with 5,000 resamples was employed to examine the significance of the indirect associations (Table 8).

**Table 8:** Chain mediation analysis of creative self-efficacy and innovation literacy among undergraduate nursing students (*N* = 337).

Variable	Active learning state		Social capital		Innovation literacy	
	β (95% CI)	*t*	β (95% CI)	*t*	β (95% CI)	*t*
Creative self-efficacy	0.594 (0.449, 0.604)	13.404***	0.162 (0.066, 0.301)	3.063**	0.315 (0.224, 0.373)	7.928***
Active learning state	—	—	0.499 (0.506, 0.771)	9.464***	0.373 (0.306, 0.492)	8.458***
Social capital	—	—	—	—	0.270 (0.159, 0.293)	6.614***
Sex	−0.003 (−0.139, 0.131)	−0.056	−0.118 (−0.399, −0.068)	−2.778**	0.004 (−0.098, 0.110)	0.116
Grade	−0.048 (−0.080, 0.023)	−1.074	0.029 (−0.042, 0.085)	0.675	−0.042 (−0.066, 0.012)	−1.344
Residence	−0.043 (−0.167, 0.057)	−0.968	−0.037 (−0.199, 0.076)	−0.874	−0.081 (−0.197, −0.026)	−2.575*
Class leader status	−0.058 (−0.189, 0.039)	−1.291	−0.083 (−0.277, 0.003)	−1.921	−0.014 (−0.107, 0.068)	−0.449
R	0.613		0.654		0.828	
R^2^	0.376		0.428		0.686	
F	39.811***		41.176***		102.704***	

*p < 0.05; **p < 0.01; ***p < 0.001.

**Figure 2:**
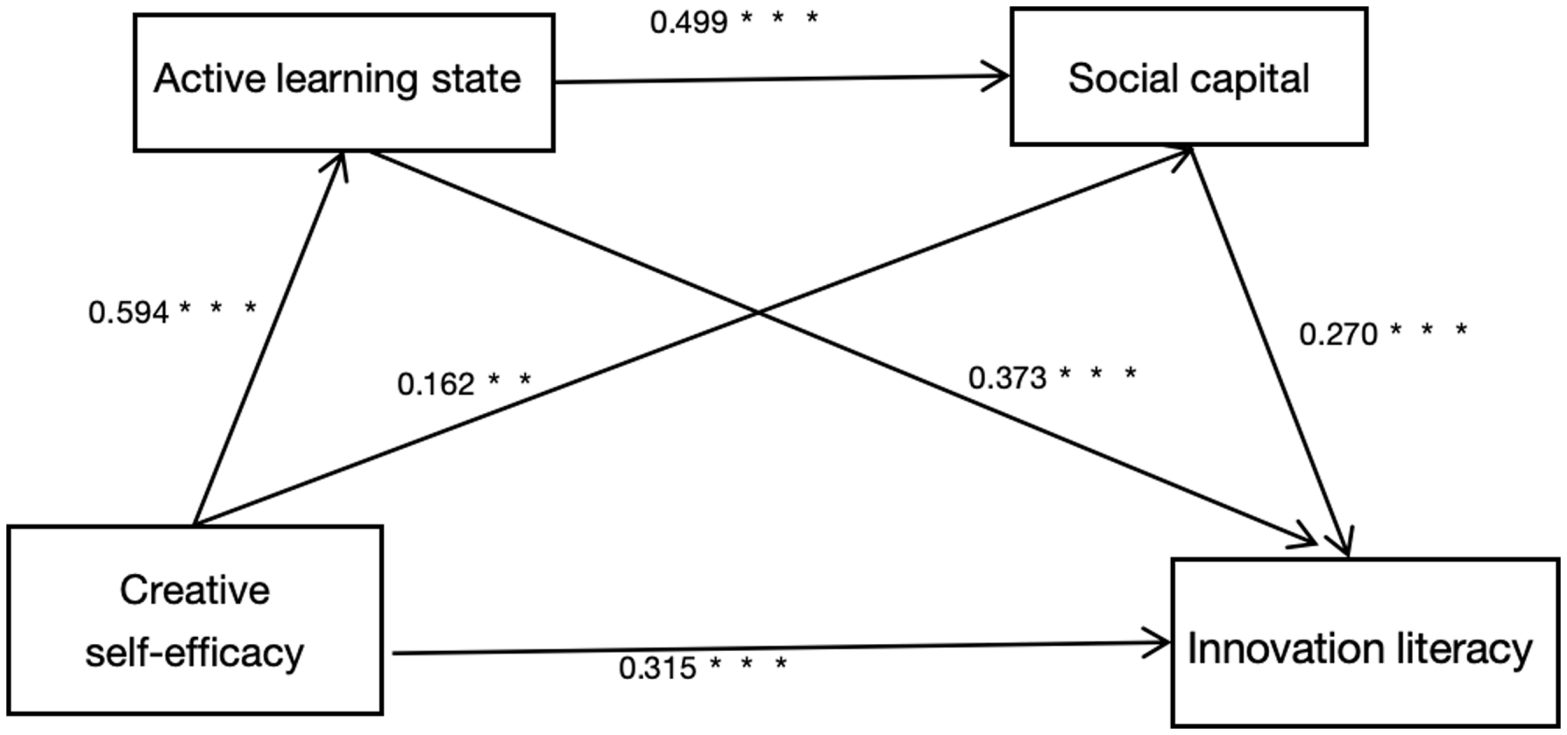
A sequential correlational framework describing the association of creative self-efficacy with innovation literacy among undergraduate nursing students. Active learning state and social capital function as sequential intermediate variables in this association.

Results for total, direct, and indirect associations are presented in Table 9 and Figure 2. The 95% bias-corrected confidence intervals (CIs) for all indirect pathways excluded zero (all *p* < 0.001), consistent with significant intermediate associations. Specifically, active learning state showed a significant intermediate association between CSE and innovation literacy (association = 0.221, 33.48% of the total association; 95% CI: 0.139, 0.313). These findings are consistent with H2. Social capital also showed a significant intermediate association (association = 0.044, 6.67% of the total association; 95% CI: 0.000, 0.090). A significant sequential intermediate association was observed through active learning state and social capital (association = 0.080, 12.12% of the total association; 95% CI: 0.034, 0.119). These findings are consistent with H3.

**Table 9:** Mediation association and confidence intervals (*N* = 337).

Mediation effect	Effect size	Proportion of total effect	95%CI (LL, UL)
Total indirect effect	0.345	53.79%	(0.249, 0.426)
Creative self-efficacy → Active learning state → Innovation literacy	0.221	33.48%	(0.139, 0.313)
Creative self-efficacy → Social capital → Innovation literacy	0.044	6.67%	(0.000, 0.090)
Creative self-efficacy → Active learning state → Social capital → Innovation literacy	0.080	12.12%	(0.034, 0.119)
Direct effect			
Creative self-efficacy → Innovation literacy	0.315	47.73%	(0.224, 0.373)
Total effect			
Creative self-efficacy → Innovation literacy	0.660	—	(0.551, 0.701)

Tips: CI, confidence interval; LL, lower limit; UL, upper limit.

The total indirect association was 0.345 (53.79% of the total association), while the direct association of CSE with innovation literacy was 0.315 (47.73% of the total association), with a total association of 0.660. All 95% CIs for the indirect associations excluded zero, consistent with robust sequential intermediate associations.

## 5 Discussion

This study aimed to examine the mediating associations of active learning state and social capital in the relationship between creative self-efficacy and innovation literacy. The findings describe patterns relevant to the development of teaching intervention programs in nursing undergraduate education and are consistent with the cultivation of innovation literacy and self-efficacy among nursing undergraduates.

### 5.1 Current status of creative self-efficacy and innovation literacy among undergraduate nursing students

The results showed that creative self-efficacy and innovation literacy among undergraduate nursing students were at moderate-to-high levels, a pattern consistent with previous research (38). However, this result should be interpreted cautiously, as moderate-to-high levels may reflect lenient evaluation criteria or students’ self-presentation preferences. Because self-report scales were used, the results may be subject to recall bias and selection bias, potentially leading to inflated overall scores and constrained upper bounds for mediating association estimates. As the digital transformation of healthcare systems continues, cultivating nursing informatics competence has become a priority in education (39). This study found that creative self-efficacy showed balanced development, whereas innovation literacy exhibited a marked structural imbalance: strong-tie resources were adequate, but weak-tie resources were insufficient. From the perspective of social network theory, weak ties are critical for accessing novel information and breaking cognitive inertia. This pattern suggests that current nursing innovation education is characterized by an “internally cohesive but externally sparse” structural contradiction, with excessive reliance on internal collaborative environments and insufficient attention to cross-boundary social network cultivation. Even if learning motivation, cognitive levels, and external environments are integrated to build a collaborative development ecosystem (18), without prioritizing the strengthening of weak-tie deficits, students’ innovation literacy may remain at incremental improvement, with limited potential for breakthrough innovation. Therefore, nursing innovation education should not focus solely on maintaining moderate-to-high scores but should target cross-boundary social network cultivation to support comprehensive innovation literacy development.

### 5.2 Group differences in innovation literacy among undergraduate nursing students

Grade, place of origin, and class leader status showed significant associations with innovation literacy, a pattern consistent with existing research (40, 41). However, the mechanisms underlying these differences merit further examination. Lower-grade students demonstrated lower innovation literacy; previous research attributed this to a longer adaptation period (41). A more fundamental explanation is that innovation courses in higher education are predominantly general education in form, lacking nursing-specific contextual embedding, which may limit the motivation of lower-grade students. Students from rural backgrounds showed significantly lower innovation literacy compared with urban students (37). Attributing this simply to “teacher quality gaps” fails to address the core issue: the differential intergenerational transmission of social capital. Rural students face challenges in accessing innovation-related information and opportunities through family and early social networks, suggesting that simply increasing curricular resources may have limited effects. Class leader status showed a positive association with innovation literacy (42). This association does not demonstrate that leadership status directly enhances innovation literacy; it is also possible that students with higher innovation literacy are more likely to be selected as class leaders. Nevertheless, this pattern suggests that interpersonal resources associated with leadership experience represent a key mechanism, and extending such resources to all students may be warranted.

### 5.3 Sequential associational mechanisms between creative self-efficacy and innovation literacy

This study found a significant positive association between creative self-efficacy and innovation literacy among undergraduate nursing students, consistent with domestic and international research (43). However, existing research has focused predominantly on engineering, management, or general education; the characteristics of nursing education—high-risk, ethics-intensive, and normative—may attenuate the direct association between creative self-efficacy and innovation literacy. In this study, although the two variables showed a significant positive correlation, the correlation coefficient did not reach the threshold for a strong association, suggesting that unique factors in the nursing context may inhibit the translation of creative self-efficacy, such as risk-averse culture during clinical internships and normative evaluation systems, which were not directly measured in this study.

Mediation analysis showed that creative self-efficacy was linked to innovation literacy through the sequential pathway of active learning state → social capital, with this sequential association accounting for 12.12% of the total association; the independent association of active learning state accounted for 33.48%. However, the independent association of social capital accounted for 6.67%, with its 95% CI including 0, indicating that this pathway did not reach statistical significance. The findings suggest that social capital did not function as an independent intermediate variable in the association between creative self-efficacy and innovation literacy; its role appeared contingent on active learning state. In other words, the significance of the sequential pathway does not imply that social capital independently mediated this association but rather reflects that active learning drives social capital accumulation. Because active learning represents a cognitive process under students’ own control, it is characterized by high efficiency and low cost. Social capital, in contrast, requires frequent coordination with others, which may be difficult to fully realize under the conditions of dense nursing curricula and dispersed clinical internships. Therefore, teaching might prioritize problem-based learning and reflective writing as strategies that elicit active learning, rather than indiscriminately increasing group collaboration. Moreover, social capital appears to function only after active learning has been activated; the focus of educational interventions may be the integration of both, initiated through triggering active learning, followed by building social capital platforms.

Interpreted through the SECI knowledge transformation model, the sequential mechanism can be understood as follows: First, students with high creative self-efficacy are more inclined to share perspectives and engage in collaborative inquiry within team settings, corresponding to the socialization stage. Second, an active learning state prompts students to organize personal experiences and express independent viewpoints, facilitating the transformation of tacit knowledge into explicit knowledge, corresponding to the externalization stage. Third, the accumulation of social capital supports students in integrating peer, instructor, and interdisciplinary resources, systematizing fragmented explicit knowledge, corresponding to the combination stage. Finally, students achieve internalization through continuous practice and reflection, thereby comprehensively enhancing innovation literacy (43–45). Nevertheless, the application of the SECI model requires caution. Its fundamental limitation lies in knowledge type mismatch. The “socialization” in SECI corresponds to procedural knowledge, whereas “creative self-efficacy” in this study is declarative belief; analogizing belief validation to tacit knowledge socialization involves conceptual drift. Furthermore, the model assumes linear progression through four stages, whereas nursing undergraduates’ learning processes are more likely to be nonlinear and iterative; cross-sectional data cannot capture this dynamic recursion, so SECI functions here primarily as an explanatory framework.

Correlational analysis showed a significant positive association between social capital and innovation literacy, consistent with research on Korean registered nurses (46). However, mediation analysis showed that the independent association of social capital was not significant (6.67%, CI including zero), suggesting that its positive association must be realized through the prior activation of active learning. Excessive reliance on homogeneous social capital may limit heterogeneous knowledge input (44, 45); in this study, social capital results showed that strong and weak ties demonstrated complementary functions. Strong ties provide a secure context, and weak ties provide heterogeneous stimulation, but both appear contingent on active learning state. Simply building platforms to accumulate social capital may be insufficient in teaching practice, and strategies combining active learning triggers and social capital activation may be warranted, while remaining alert to the risk of social capital enclosure in the absence of active learning.

### 5.4 Critical reflection

This study used a cross-sectional design; the temporal sequence assumptions of mediating associations were based on theoretical derivation rather than empirical verification. Reverse pathways are equally plausible: innovation literacy may enhance creative self-efficacy through achievement, active learning may strengthen creative self-efficacy, and social capital may facilitate active learning. If bidirectional relationships exist simultaneously, traditional mediation estimates may yield bias, including overcontrol bias. These endogeneity issues cannot be fully resolved through statistical methods; future research may employ cross-lagged panel designs or randomized intervention experiments to establish stronger bases for causal inference.

## 6 Implications

### 6.1 Theoretical Implications

This study integrated social cognitive theory and the SECI knowledge creation model to examine sequential intermediate associations of active learning state and social capital in the context of creative self-efficacy and innovation literacy. Specifically, this study described potential applicability boundaries of the SECI model at the individual level—the combination stage (social capital) appeared unable to function independently of the externalization stage (active learning). This pattern suggests a refinement of the implicit assumption that the four SECI stages constitute necessary sequential paths and may inform subsequent longitudinal and intervention research.

### 6.2 Practical implications

The findings indicated that the independent intermediate association of social capital with innovation literacy was not statistically significant; its role appeared contingent on the prior activation of active learning state. Additionally, weak-tie resources represented a structural deficit in nursing innovation literacy. The following strategies are consistent with these findings:

1. Active Learning Operationalization: Problem-based learning cases might focus on risk decision-making and resource constraint issues in nursing contexts, with core courses potentially embedding 2–3 complete inquiry-based learning cycles (based on course density estimates from this sample). Reflective writing could be combined with clinical internships, with students potentially recording one nursing pain point and improvement idea weekly and receiving structured feedback from instructors.
2. Actionable Social Capital Interventions: Each semester could include a cross-boundary innovation day, introducing weak-tie roles such as clinical engineers and patient representatives. Rotating leadership and cross-team collaboration rules might be implemented to avoid concentration of collaborative resources among class leaders. For rural and lower-grade students, innovation social capital workshops and peer mentor matching could be added.
3. Curriculum and Evaluation Reconstruction: Active questioning frequency and cross-group collaboration records might be incorporated into formative assessment (10–15%). A 2-credit innovation practice course could be offered, with problem-based learning and weak-tie input as core components and scheduled separately from clinical internships. Given the self-report design of this study, future research might explore a mixed evaluation model combining behavioral indicators and self-report scales, which could be used in combination to track innovation literacy development.

## 7 Limitation

First, this study used a cross-sectional design, limited to the exploration of associations among variables. This design cannot establish causal directions, temporal sequences, or exclude reverse or reciprocal interactions between variables, which limits the explanatory scope of the findings in this sample. Second, all data were collected through self-report scales, which are susceptible to social desirability bias, recall bias, and common method variance. Such biases are associated with potentially inflated association estimates between variables and may affect the objectivity of the results. Although this study implemented procedural measures including anonymous completion, staggered distribution, differentiated instructions, and embedded lie-detection items, combined with Harman’s single-factor test for statistical control, these approaches are consistent with partial mitigation of the above biases, the inherent limitations of self-report methods cannot be fully eliminated. Third, the sample was restricted to undergraduate nursing students from a single medical university in a specific geographic region of China. This sampling strategy limits generalizability to nursing students in other institutions, regions, or countries with different educational systems, cultural contexts, and training programs. In addition, the analytical approach used is sensitive to sample size and the number of measurement items. The relatively small sample and numerous observed items are associated with potential effects on model fit and parameter stability, which may be relevant to the reliability of the results.

Future research might employ longitudinal or cross-lagged designs, which could provide stronger bases for examining causal relationships and dynamic changes among variables. Multiple sources of information, such as other-ratings and behavioral performance, could be considered to address methodological biases associated with self-reports. Meanwhile, multi-center and large-sample sampling could enhance representativeness and generalizability, supporting more rigorous examination of the stability and applicability of the proposed model.

## 8 Conclusion

This study found moderate levels of innovation literacy. Creative self-efficacy, active learning state, and social capital showed positive associations with innovation literacy. The sequential intermediate variables were active learning state and social capital. Creative self-efficacy was related to innovation literacy via active learning state and social capital as sequential intermediate variables.

## Data Availability

The raw data supporting the conclusions of this article will be made available by the authors, without undue reservation.
